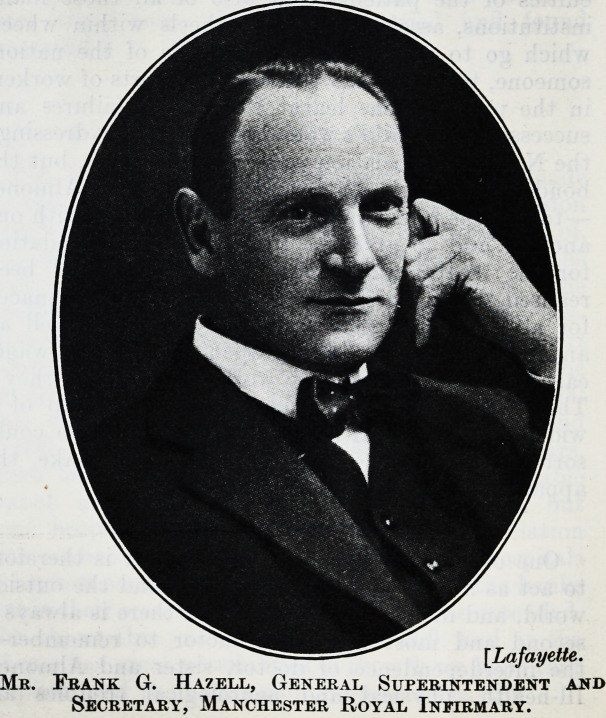# Hospital Officers Conference

**Published:** 1924-06

**Authors:** 


					178 THE HOSPITAL AND HEALTH REVIEW June
THE HOSPITAL OFFICERS' CONFERENCE.
The first annual conference of the Incorporated
Association of Hospital Officers is being held at the
Central Hall, Westminster, as we go to press. The
subjects of discussion include "Voluntary Hospitals
and Paying Guests," by Mr. H. J. Waring, senior
surgeon to St. Bartholomew's Hospital; "Accounting
inJRelation to Hospital Administration." by Mr. J. E.
Stone, St. Thomas's, a summary of which we print on
another page; " The Changing Character of the
Voluntary System," by Mr. C. A. Mason, secretary
of the Midland Eye Hospital, Birmingham; and
" Maintenance," by Mr. F. B. Hazell, secretary
of the Manchester Royal Infirmary. The Association
was founded in 1202.
{Elliot and Fry.
Mr. H. J. Waring, C.B.E., M.S., F.R.C.S., Vice-Chancellor
of London University.
[Vandyk.
Mr. H. L. Eason, C.B., C.M.G., M.D., M.S., President
OF THE HOSPOITAL OFFICERS' ASSOCIATION.
Mr. 0. A. Mason, General Superintendent an? Secretary,
Birmingham and Midland Eye Hospital.
Mr. 0. A. Mason, General Superintendent and Secretary,
Birmingham and Midland Eye Hospital.
[Lafayette.
Mb. Frank G. Hazell, General Superintendent and
Secretary, Manchester Royal Infirmary.
[Lafayette.
Mr. Frank G. Hazell, General Superintendent and
Secretary, Manchester Royal Infirmary.

				

## Figures and Tables

**Figure f1:**
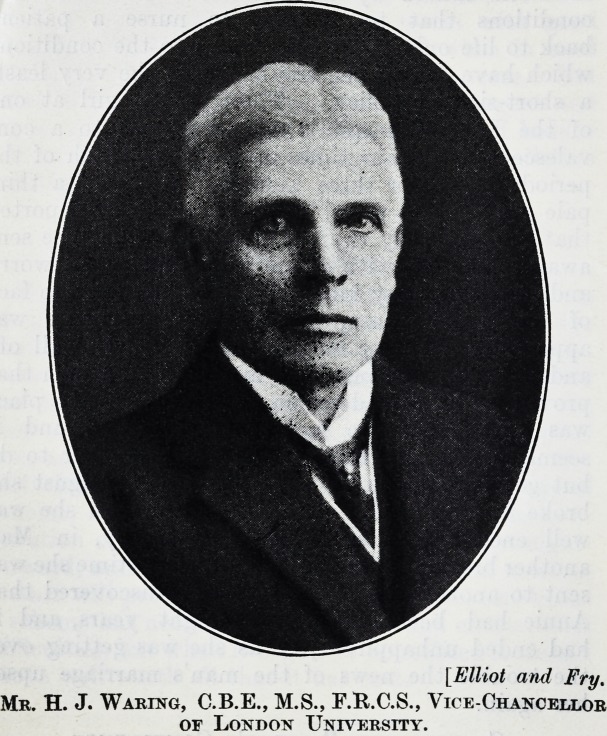


**Figure f2:**
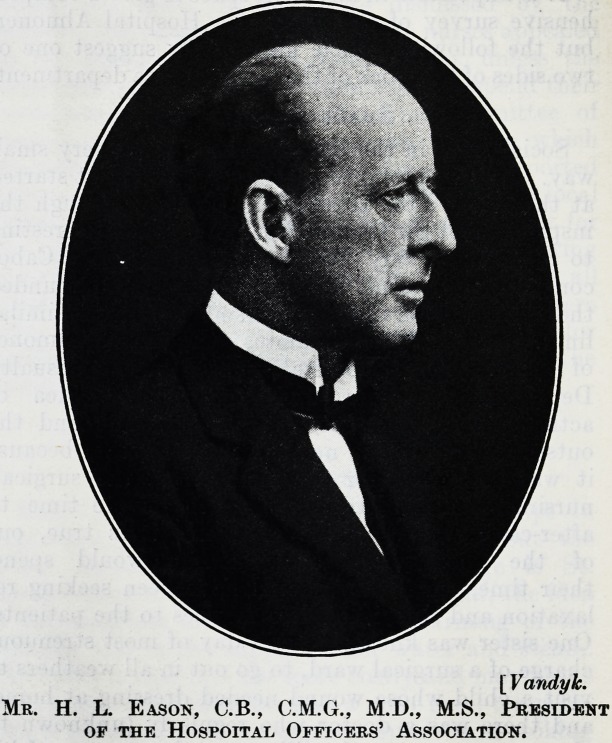


**Figure f3:**
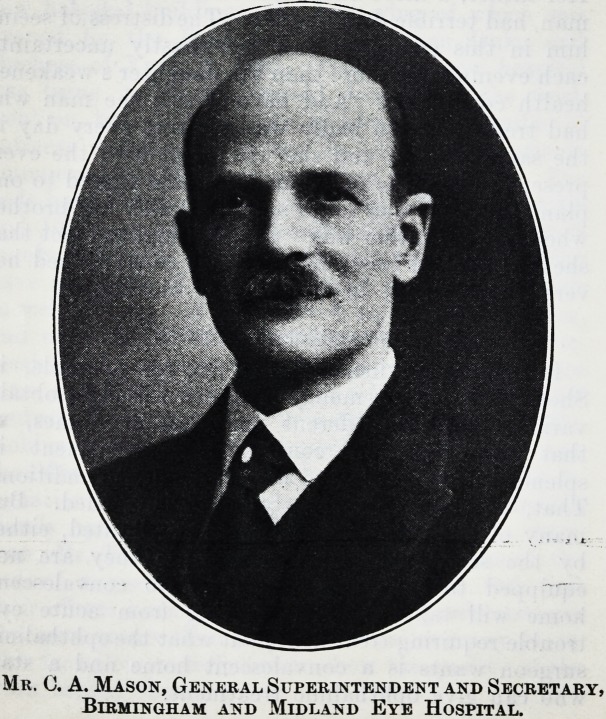


**Figure f4:**